# Apigenin impedes cell cycle progression at G_2_ phase in prostate cancer cells

**DOI:** 10.1007/s12672-022-00505-1

**Published:** 2022-06-07

**Authors:** Su Su Thae Hnit, Mu Yao, Chanlu Xie, Ling Bi, Matthew Wong, Tao Liu, Paul De Souza, Zhong Li, Qihan Dong

**Affiliations:** 1 Chinese Medicine Anti-cancer Evaluation Program, Greg Brown Laboratory, Central Clinical School and Charles Perkins Centre, The University of Sydney, Sydney, NSW Australia; 2 Department of Endocrinology, Royal Prince Alfred Hospital, Sydney, NSW Australia; 3 School of Natural Sciences, Faculty of Science and Engineering, Macquarie University, Sydney, NSW Australia; 4 Department of Oncology, Yueyang Hospital of Integrated Traditional Chinese and Western Medicine, Shanghai University of Traditional Chinese Medicine, Shanghai, China; 5 Children’s Cancer Institute Australia for Medical Research, Sydney, NSW Australia; 6 Centre for Childhood Cancer Research, UNSW Medicine, Sydney, Australia; 7 School of Medicine, Western Sydney University, Sydney , Australia; 8 Dongzhimen Hospital, Beijing University of Chinese Medicine, Beijing, China; 9 Faculty of Medicine and Health, University of Sydney, 2006 Camperdown, NSW Australia; 10 Beijing University of Traditional Chinese Medicine, 201203 Beijing, China

**Keywords:** Apigenin, G_2_ phase, Phospho-histone H3, Prostate

## Abstract

As a natural flavone, apigenin is abundantly present in vegetables, fruits, oregano, tea, chamomile, wheat sprout and is regarded as a major component of the Mediterranean diet. Apigenin is known to inhibit proliferation in different cancer cell lines by inducing G_2_/M arrest, but it is unclear whether this action is predominantly imposed on G_2_ or M phases. In this study, we demonstrate that apigenin arrests prostate cancer cells at G_2_ phase by flow cytometric analysis of prostate cancer cells co-stained for phospho-Histone H3 and DNA. Concurrently, apigenin also reduces the mRNA and protein levels of the key regulators that govern G_2_-M transition. Further analysis using chromatin immunoprecipitation (ChIP) confirmed the diminished transcriptional activities of the genes coding for these regulators. Unravelling the inhibitory effect of apigenin on G_2_-M transition in cancer cells provides the mechanistic understanding of its action and supports the potential for apigenin as an anti-cancer agent.

**Graphical Abstract:**

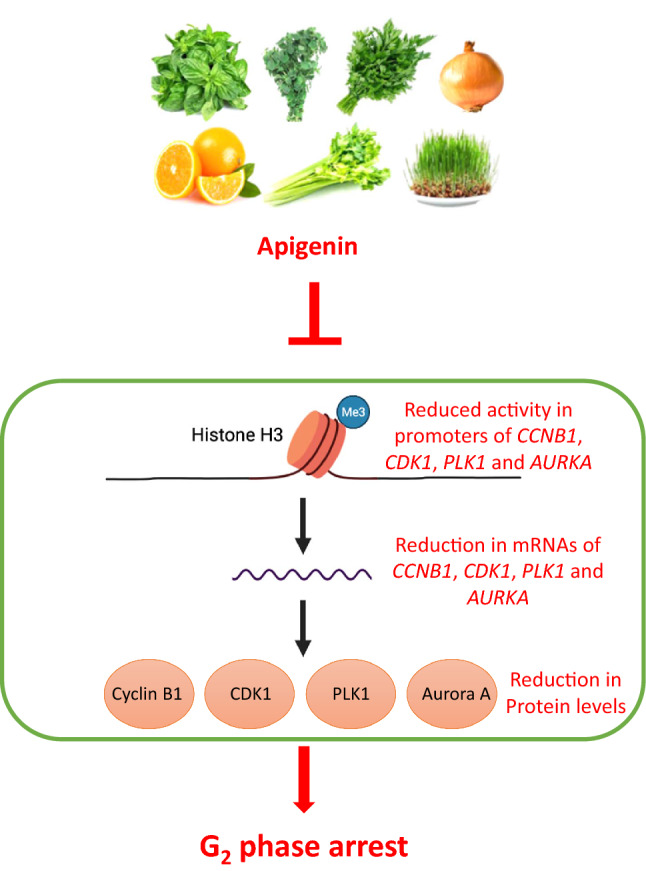

**Supplementary Information:**

The online version contains supplementary material available at 10.1007/s12672-022-00505-1.

## Introduction

Prostate cancer is the most prevalent non-skin cancer in men and remains the leading cause of cancer-related death [1, 2]. Although progress has been made in the development of new treatment modalities, management of patients with advanced disease remains challenging [3]. In recent years, there has been increasing interest in finding dietary components that have potential to prevent and control prostate cancer [4]. A natural flavone, apigenin is present in a variety of food items [5, 6] and exhibits numerous beneficial effects including anti-oxidation [7], anti-inflammatory [8], anti-mutagenic [9], anti-cancer [10] and anti-microbial activities [11]. It is a major component of the Mediterranean diet, which has been shown to be associated with lower incidence of many human diseases including cancer [12]. Previous studies have demonstrated that apigenin exerts its substantial inhibitory effect on proliferation of cancer cells from breast [13], pancreatic [14], renal [15], liver [16], lung [17], colon [18, 19] and prostate [20] cancer. The inhibition of tumour growth by apigenin in diverse animal models were also reported [21–24]. Further studies have established that the anti-proliferative action of apigenin is related to an induction of G_2_/M arrest in cancer cell cycle [13–15, 18, 19, 21, 22, 25]. However, it is unclear if the arresting action is exerted on G_2_ phase, or M phase because each action imposes a different biological impacts on cancer cells [26]. Targeting cancer cell mitosis represented by taxol remains a cornerstone in combating cancer diseases [27]. If apigenin affects transition from G_2_ to M phase, a combination of apigenin with a mitosis-targeting agent is worthy considering as it could impede cell cycle progression of cancer cells at both G_2_ and M phases. The aim of the study was to further dissect the G_2_/M arresting action of apigenin. We report that apigenin affects prostate cancer cells at G_2_ phase rather than at M phase with concomitant down-regulation of the regulators that govern G_2_-M transition at the transcriptional level including CDK1, CyclinB1, Polo-like kinase 1 (PLK1) and Aurora A [28].

## Materials and methods

### Cell lines

Lymph node metastasis-derived prostate cancer cell line, LNCaP (CRL- 1740; American Type Culture Collection), and bone metastasis-derived prostate cancer cell line, PC-3 (CRL-1435; American Type Culture Collection), were grown in RPMI 1640 supplemented with 10% v/v fetal bovine serum (AusGeneX), penicillin at 100U/mL (Invitrogen) and streptomycin at 100 µg/mL (Invitrogen). The cells were cultured at 37 °C in an incubator providing a humidified environment in the presence of 5% CO_2_/95% air.

### Preparation of Apigenin

Apigenin (B20981, Lot Y27A6C1, 20MG), CAS 520-36-5, was purchased from Shanghai Yuanye Biotechnology. Purity was above 98% through LC-UV analysis. Apigenin was first dissolved in DMSO at concentration of 100 mM as stock and stored at 4 °C. On the day of experiment, further dilution of the stock was performed to obtain the desired working concentrations of apigenin with complete RPMI 1640. Simultaneously, DMSO was diluted with the medium at 0.1% to serve as vehicle control (0 µM) as described previously [29].

### SYBR Green assay

The effect of apigenin on the proliferation of prostate cancer cells were assessed by SYBR Green assay. LNCaP (2000 cells/well) and PC-3 (12,000 cells/well) cells were seeded in 96-well plates and cultured overnight. On the following day, the culture medium was gently removed, and the cells were treated with apigenin at different concentrations (0 to 50 µM) in fresh medium. The spare plates of cells were used for baseline measurement of DNA content immediately prior to treatment for the purpose of calculating the net change in DNA content over the experimental period and stored at -80◦C. Seventy-two hours (72 h) after treatment, the medium was gently aspirated and 100 µL of lysis buffer containing SYBR Green I (S-7563, Invitrogen) at 1:10,000 dilution was added. The lysis buffer was made up of 9 portions of buffer A (10 mM Tris-HCl, pH 7.5 and 2 M NaCl) and 1 portion of buffer B (100 mM Tris-HCl, pH 7.5, 50 mM disodium EDTA and 1% Triton X-100). The cells were then lysed in the dark for 3 h on a plate shaker. The frozen cells used for baseline control were thawed at room temperature, lyzed in the same manner and transferred to the unused wells of treatment plate. The fluorescence intensity of SYBR Green-labeled DNA was measured using a plate reader (FLUOstar Omega, BMG Labtech) with excitation at 485/20 nm and emission at 528/20 nm as previously described [30, 31]. The growth inhibition (GI) is calculated using the formula: [(FI of control − FI of baseline) − (FI of treated − FI of baseline)]/(FI of control − FI of baseline) × 100%. FI is fluorescence intensity from SYBR green [32].

### Flow cytometric analysis

Propidium iodide (PI) staining was used to analyze cell cycle phase distribution. LNCaP (2.5 × 10^5^ cells/well) and PC-3 (1.2 × 10^5^ cells/well) cells were seeded in T25 flask and cultured overnight prior to treatment with apigenin at different doses (0 to 25 µM) for 72 h. Cells were harvested and stained with PI as described previously [30, 31]. To differentiate cells at G_2_ phase that contain 4n DNA content but are negative for phospho-Histone H3 from mitotic cells that also hold 4n DNA content but are positive for phospho-Histone H3 [33], the harvested cells were fixed with 70% ethanol and blocked with 1% BSA in PBS. Thereafter, the cells were sequentially labeled with antibody to phospho-Histone H3 (9701, Cell Signaling Technology) and a secondary antibody conjugated with AlexaFluor488 (A11008, Life Technology) diluted in PBS containing 1% BSA in the presence of 0.1% saponin prior to PI staining. As a reference, the cancer cells were treated with nocodazole at 0.8 µg/mL for 16 h to enrich cells at M phase and then subjected to the same labeling procedure. Saponin was exploited to enhance the intracellular staining by permeabilization of the fixed cells [34]. Flow cytometric data on the labeled cells were then acquired by using a flow cytometer (FACS Calibur, BD Biosciences) and analyzed by using FlowJo software [28].

### Immunoblotting

LNCaP and PC-3 cells were treated in T25 flasks at different doses (0 to 25 µM) of apigenin for 72 h and cell lysates were prepared with RIPA-buffer (R0278, Sigma-Aldrich) supplemented with protease inhibitor cocktail (11,836,145,001; Roche) as described previously [30, 31]. Cells in the lysis buffer were sonicated in 1.5-mL Eppendorf tubes on ice. The cell lysates were then centrifuged at 12,000*g* for 1 min and resultant supernatants were collected and stored at -80 °C until use. To detect proteins of interest, proteins were separated on SDS-polyacrylamide gel electrophoresis followed by transfer to Polyvinylidene fluoride (PVDF) membranes (ISEQ00010, Immobilon). Membranes were incubated with 2% skim milk in TBST (TBS containing 0.5% Tween 20, pH 7.5) for 30 min prior to labeling with primary antibodies against proteins of interest at 4 °C overnight. Primary antibodies against: Cyclin B1 (SC-752) and GAPDH (SC-137,179) were purchased from Santa Cruz Biotechnology; CDK1 (9116), PLK1 (4513), Aurora A (12,100) were obtained from Cell Signaling Technology. On the following day, membranes were washed with TBST and incubated with horse radish peroxidase-conjugated anti-mouse (A4416, Sigma-Aldrich) or anti-rabbit (A0545, Sigma-Aldrich) secondary antibodies for 2–3 h at room temperature. Thereafter, the membranes were washed with TBST and in the presence of Clarity Western ECL substrate (170–5060, Bio-Rad) the immuno-labeled proteins were captured by BioRad ChemiDoc MP system (Universal Hood II, Bio-Rad) equipped with Image Lab (version 6.0.0).

### Reverse transcription quantitative PCR (RT-qPCR)

LNCaP and PC-3 cells were treated with apigenin in 6-well plates at different doses (0 to 25 µM) for 72 h prior to assessing gene expressions of interest by RT-PCR. RNA in the experimental cells was extracted with Purelink RNA Mini Kit (12,183,018 A, Life Technologies) and the first strand cDNA was generated with the iScript™ cDNA Synthesis Kit (BioRad). The cDNA was then mixed with each pair of primers and a SensiMix Kit (QT650-05, Bioline) and PCR reaction was initiated at 95ºC for 10 min and generated by subsequent 45 thermocycles at 95 ºC for 15 s, at 60ºC for 15 s and at 72 ºC for 15 s. The primer sequences are described in Additional file 1: Table S1.

### Chromatin immunoprecipitation (ChIP)

PC-3 cells were treated with apigenin at 0 or 25 µM in T150 flasks for 48 h and harvested for ChIP performed with immunoprecipitation assay kit (17–295, Merck) and an antibody to H3K4me3 (ab8580, Abcam) or isotype IgG (SC-2027, Santa Cruz Biotechnology) as a negative control. Both immunoprecipitated DNA and DNA input for assay quality control were then analyzed by quantitative PCR. The resultant data were calculated and expressed as fold change of the values with the primary antibody relative to those with the isotype IgG. The primer sequences were listed in Additional file 1: Table S2.

### Statistical analysis

The statistical software NCSS (v12.0; Kaysville) and Prism (GraphPad) version 9.3.1 (350) were used for analysis. One-Way ANOVA was implemented to determine if there was any significant difference among treatment doses. Fisher’s LSD multiple comparison test was used to determine group differences (p < 0.05).

## Results

### Apigenin suppressed cell proliferation in prostate cancer cells

**Fig. 1:**
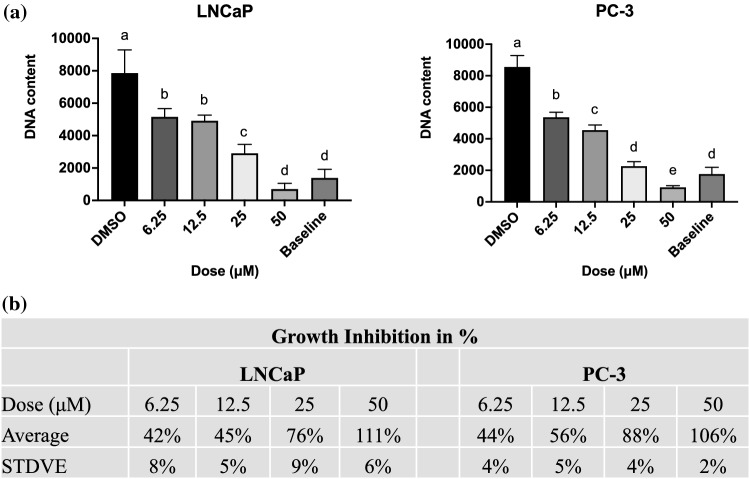
Apigenin inhibited the DNA synthesis in prostate cancer cells. **a** LNCaP and PC-3 cells were treated with apigenin at indicated doses for 72 h and harvested for SYBR Green assay. DNA contents were expressed as the mean ± SD of three independent experiments. Means without a common letter differ, p < 0.05. **b** The percentage of growth inhibition by apigenin in two prostate cancer cell lines were calculated and showed as mean ± SD

The anti-proliferating efficacy of apigenin in prostate cancer cells was first assessed by SYBR Green assay to measure the net change in DNA content in LNCaP and PC-3 cells over experimental period. Compared with vehicle control (in the absence of apigenin), apigenin reduced the net gain of DNA content in prostate cancer cells in a dose-dependent manner (Fig. 1).

### Apigenin arrested prostate cancer cells at G_2_ phase

**Fig. 2:**
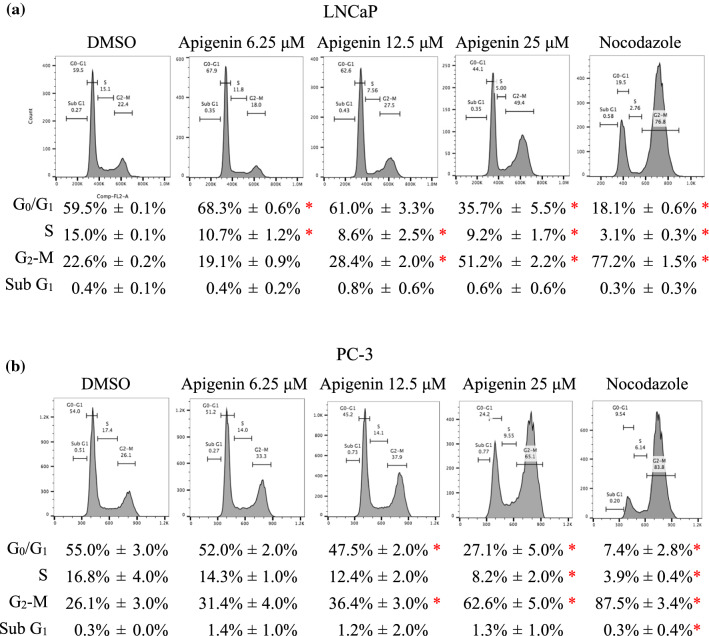
Apigenin increased prostate cancer cells at G_2_/M phase. **a** LNCaP and **b** PC-3 cells were treated with apigenin at indicated doses for 72 h and stained with PI for cell cycle analysis. Cells treated with nocodazole (0.8 µg/mL) for 16 h were used as a reference. Representative histograms of PI staining were presented. Cell cycle distribution in percentage was expressed as the mean ± SD from three independent experiments. *p < 0.05 compared to Control

**Fig. 3:**
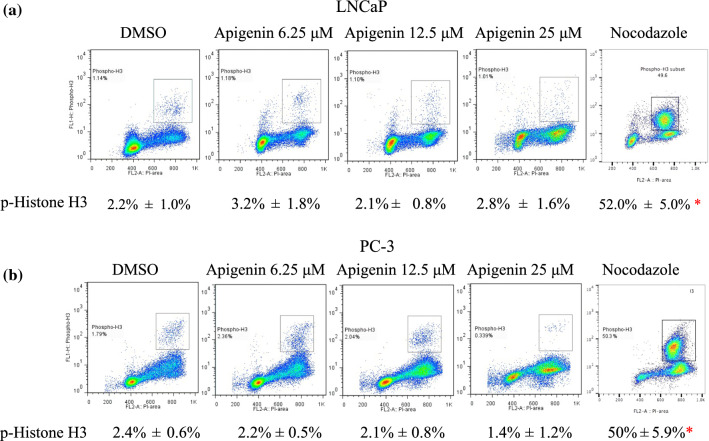
Apigenin increased prostate cancer cells at G_2_ phase. **a** LNCaP and **b** PC-3 cells were treated with apigenin at indicated doses for 72 h and labeled with both an antibody against phospho-Histone H3 and PI for detection of miotic cells. To enrich mitotic cells as reference, the cancer cells were treated with nocodazole (0.8 µg/mL) for 16 h. The percentages of miotic cells were expressed as the mean ± SD from three independent experiments. *p < 0.05 compared to Control

To delineate the anti-proliferating effect of apigenin from the perspective of cell cycle progression, the prostate cancer cells were treated for 72 h and stained with PI for cell cycle analysis by flow cytometry. Compared with vehicle control, apigenin increased the cancer cells at G_2_/M phase in a dose dependent fashion (Fig. 2). To determine whether apigenin arrested the cancer cells at G_2_ or M phase, the cancer cells were treated for 72 h and then co-stained with an antibody to phospho-Histone H3 at Ser10 (a mitotic marker) and PI for further flow cytometric analysis. At the same time, nocodazole was used to arrest the cancer cells at M phase as a reference. Compared to the control, apigenin did not increase the cancer cells at M phase, which was in contrast with what was seen in nocodazole-treated cells. These results illustrate that apigenin arrested the cancer cells at G_2_ phase instead of at M phase (Fig. 3).

### Apigenin transcriptionally reduced the regulatory proteins governing G_2_ to M transition

**Fig. 4:**
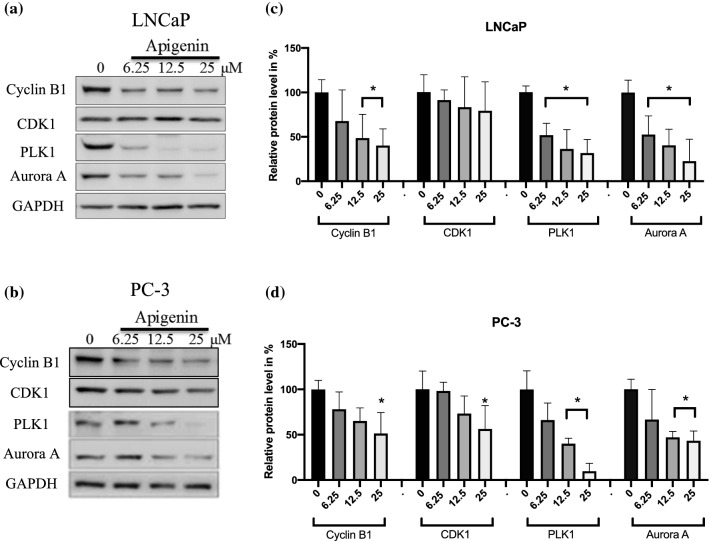
Apigenin down-regulated the regulators governing G_2_ to M transition at protein levels in prostate cancer cells. **a** LNCaP and **b** PC-3 cells were treated with a dose range of apigenin (0–25 µM) for 72 h and harvested for immunoblotting. The protein levels were quantified using Bio-Rad image lab application and the data were shown as the mean ± SD from three different experiments, **c** and **d**. *p < 0.05 compared to Control

**Fig. 5:**
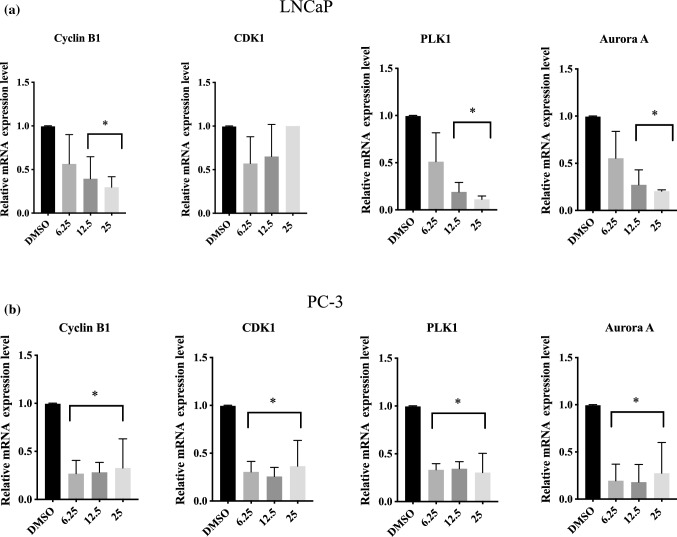
Apigenin reduced mRNA level of the regulators that govern G_2_ to M transition in prostate cancer cells. **a** LNCaP and **b** PC-3 cells were treated with a dose range of apigenin (0–25 µM) for 72 h and harvested for RT-PCR. The relative mRNA expression levels from were calculated using REST 2009 software The data from were shown as the mean ± SD from three different experiments. *p < 0.05 compared to control

**Fig. 6:**
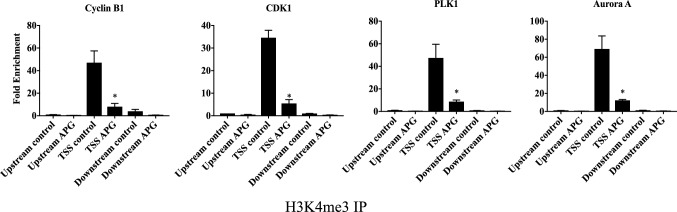
Apigenin suppressed the transcription of the regulators that govern G_2_ to M phase transition. PC-3cells were treated with 0 µM or 25 µM of apigenin for 48 h and harvested for ChIP assay with anti-H3K4me3 antibody or rabbit IgG. The immuno-precipitated DNA samples were analysed by real-time PCR with primers targeting a region around transcription start site (TSS) and two negative control regions (the regions upstream and downstream to TSS). The data were shown as the mean ± SD from three different experiments. *p < 0.05 compared to control

To study the mode of action of apigenin in prostate cancer cells, protein and mRNA levels of the essential regulators for G_2_ to M transition were assessed. Apigenin reduced CyclinB1, PLK1 and Aurora A in both LNCaP and PC-3 cells at both protein (Fig. 4) and mRNA (Fig. 5) levels. In addition, both protein and mRNA levels of CDK1 were also reduced in PC-3 cells. To further dissect the mechanism underlying the findings, ChIP was performed to evaluate the levels of H3K4 trimethylation (H3K4me3) at promoter regions of these genes in PC-3 cells. Consistently, apigenin reduced the degree of this epigenetic modification (Fig. 6), reinforcing the notion that apigenin diminished gene expression of the regulators governing G_2_ to M transition in prostate cancer cells.

## Discussion

Apigenin is a plant flavone and naturally occurs as a 4′,5,7-trihydroxyflavone with hydroxyl groups at positions C-5 and C-7 of the A-ring and C-4′ of the B-ring. It is abundantly present in parsley, onion, celery, basil, oregano, orange, tea, chamomile and wheat sprout [5, 6] and other vegetables [35] and fruits [36–38]. Epidemiologic studies indicate that a diet rich in flavones including apigenin is associated with a reduced risk of a variety of cancers [38].

To investigate the mode of action for apigenin, numerous laboratory studies have been conducted and most frequently apigenin has been shown to arrest a number of types of cancer cells at G_2_/M phase [13–15, 18, 19, 21, 25]. To our knowledge, a further cell cycle analysis to dissect action of apigenin on the cell cycle has not been attempted. We reasoned that if apigenin acts on the G_2_ phase, one can envisage a combination of apigenin with mitosis-targeting agents (e.g., docetaxel, the most commonly used chemotherapeutic agent for the treatment of metastatic prostate cancer through stabilizing microtubules) is more effective for the treatment of advanced prostate cancer by simultaneous inhibition of cell cycle progression at both G_2_ and mitotic phases. On the other hand, if apigenin affects M phase by destabilizing microtubule, it is expected to antagonize the effect of docetaxel [39, 40].

In agreement with previous reports [10, 41, 42], apigenin in our study was shown to exert its inhibitory effect on cell proliferation through G_2_/M arrest in prostate cancer cells. To further dissect the cell cycle arrest, the cancer cells were co-stained for phospho-Histone H3 and DNA to distinguish the cells at G_2_ phase from mitotic cells [28], and subsequent flow cytometric analysis of the stained cancer cells revealed that apigenin impeded progression through G_2_ phase instead of passage through M phase. In line with our previous study [28], the underlying mechanism of the observed G_2_ arrest was reduced transcription of the essential regulators governing the G_2_-M transition, as evidenced by down-regulation of mRNAs for cyclin B1, PLK1 and Aurora A in both LNCaP and PC-3 cells. In addition, CDK1 was also down-regulated at mRNA level in PC-3 cells. Consistently, the corresponding protein levels of the regulators were also diminished. Further analysis of gene transcription activity was performed by exploiting H3K4 trimethylation (H3K4me3) at promoter regions of genes as a marker of active transcription [43, 44]. The analysis illustrated that apigenin substantially lessened the levels of H3K4me3 in the promoter regions of the genes coding for CDK1, cyclin B1, PLK1 and Aurora A, indicating repressed transcription of the corresponding genes. Considering a reduction of cyclin B1 and CDK1 by apigenin has been reported in other cancer cell lines [10], it is possible that the impeding effect imposed on G_2_-M transition by apigenin is a general mode of action in a broad range of cancer cells.

Revealing the G_2_ arresting action of apigenin through its inhibitory effect on the transcription of Cyclin B1, CDK1, PLK1 and Aurora A is instructive in contemplating the implication of our findings. Firstly, the mRNA levels of Cyclin B1, CDK1, PLK1 and Aurora A are significantly higher in primary localized prostate cancer than those in benign prostatic hyperplasia, and the levels of these regulators are further elevated in prostate cancer with metastasis compared with those in primary localized prostate cancer [28]. Secondly, higher expressions of these genes have been found to be associated with poor prognosis in the patients with cancer diseases [45–47]. Thirdly, the inhibition of these regulators as therapeutic targets with their respective inhibitors in clinical trials has commenced [48–50]. Therefore, apigenin or apigenin-enriched food may potentially synergize mitosis-targeting agents such as docetaxel for the treatment of advanced prostate cancer by targeting both G_2_ and M phases. Equally important, apigenin imposes less cytotoxicity to benign human prostate epithelial cells than counterpart cancerous cells under *in vitro* condition [51], and it suppresses prostate cancer growth without perceivable side effects in mouse models of human prostate cancer [24, 52], suggesting it has a good safety profile. However, it is also noteworthy that apigenin has a low solubility in water [53] and a low bioavailability after oral administration [54]. Accordingly, an effort to optimize formulation of apigenin has been made [55], which should help to overcome the obstacles with regard to its potential clinical applications.

In conclusion, this study further dissected the action of apigenin on the cell cycle progression in prostate cancer cells and showed that apigenin causes cell cycle arrest in G_2_ phase by repression of transcriptional activity of the genes for the regulators governing G_2_-M transition.

## Supplementary Information

**Additional file 1: Table S1.** Primer sequence for RT-PCR. **Table S2.** Primer sequence for ChIP.

## Data Availability

The datasets used and/or analyzed during the current study are available from the corresponding author on reasonable request.
