# Supplementary material for: Apigenin impedes cell cycle progression at G2 phase in prostate cancer cells
Source: Discov Oncol. 2022 Jun 7;13:44. doi: 10.1007/s12672-022-00505-1 (PMC9174405; doi:10.1007/s12672-022-00505-1)
Supplement: Supplementary file 1 — Additional file 1: Table S1. Primer sequence for RT-PCR. Table S2. Primer sequence for ChIP. [file 12672_2022_505_MOESM1_ESM.docx]

Table S1 primer sequence for RT-PCR

| Gene | Sequence |
| --- | --- |
| CCNB1 (F) | 5'–CGGGAAGTCACTGGAAACAT–3 ' |
| CCNB1 (R) | 5'–ATTCTGCATGAACCGATCAA– 3 ' |
| CDC2 (F) | 5'–GGGGTCAGCTCGTTACTCAA– 3 ' |
| CDC2 (R) | 5'–AGTGCCCAAAGCTCTGAAAA–3 ' |
| PLK1 (F) | 5'–GCCCCTCACAGTCCTCAATA– 3 ' |
| PLK1 (R) | 5'–CTGCAGCATGTCACTGAGGT– 3 ' |
| AURKA (F) | 5'–TGCACCACTTGGAACAGTTT– 3 ' |
| AURKA (R) | 5'–ACTGACCACCCAAAATCTGC– 3 ' |
| 18S (F) | 5’–CGGCTACCACATCCAAGGAA–3 ' |
| 18S (R) | 5’–GCTGGAATTACCGCGGCT– 3 ' |

F = Forward

R = Reverse

Table S2 Primer sequence for ChIP

| Oligo Name | Sequence |
| --- | --- |
| CyclinB1 Exon1 (F) | TCTGCTGGGTGTAGGTCCTT |
| CyclinB1 Exon1 (R) | AGCAGGCAGCAGCTAAGAAG |
| CyclinB1 upstream (F) | ATGAGATCCCAACCCATGAA |
| CyclinB1 upstream (R) | AACCTCCCAAAGTGCTGGTA |
| CyclinB1 downstream (F) | CCACCCCCTGTTCCTAGAAT |
| CyclinB1 downstream (R) | GGCTGAAGCAGGAGAATCAC |
| CDK1 TSS (F) | AGTCTACGGGCTACCCGATT |
| CDK1 TSS (R) | CTCCGCTCAATTTCCAAGAG |
| CDK1 upstream (F) | CAACACCTCCAGCGGTAACT |
| CDK1 upstream (R) | ATTACCCTTTGGGCCAACTT |
| CDK1 downstream (F) | GGTGTGTTCAATGCATTTCG |
| CDK1 downstream (R) | GAGGGACAAGTGGTGTTCAAA |
| PLK1 TSS (F) | AAGAGATCCCGGAGGTCCTA |
| PLK1 TSS (R) | GGCTTGAGCAGCAGAGACTT |
| PLK1 upstream (F) | CTCCCAATGAGAACCCTGAA |
| PLK1 upstream (R) | GACAAAGTCCAGGGAAACCA |
| PLK1 downstream (F) | CTGTCCTTCAATCCGTGGTT |
| PLK1 downstream (R) | GCTTGGCATTCAATCCATTT |
| AuroraA Intron 1 (F) | TTTTGTCCTCCGAGATCACC |
| AuroraA Intron 1 (R) | TAAACCGCACTCCTGCTTTT |
| AuroraA upstream (F) | AACAAATGGGATCGCTATGG |
| AuroraA upstream (R) | CTGGCCTGAGGGTCTTTGTA |
| AuroraA downstream (F) | TCATCCCTCCTACCCCTTTT |
| AuroraA downstream (R) | CTCCCAAAGTGCTGGGATTA |

F = Forward

R = Reverse

TSS = Transcription Start Site

upstream = region before the transcription start site

downstream = region after the transcription start site
